# Sodium-glucose transport protein 2 inhibitor use in diabetes and its association with tuberculosis incidence

**DOI:** 10.1186/s12916-025-04460-w

**Published:** 2025-11-17

**Authors:** Chia-Jung Liu, Kuang-Ming Liao, Chung-Han Ho, Chin-Chung Shu

**Affiliations:** 1 Department of Internal Medicine, National Taiwan University Hospital, Hsin-Chu Branch, Hsinchu, Taiwan; 2 Graduate Institute of Clinical Medicine, College of Medicine, National Taiwan University, Taipei, Taiwan; 3 Department of Internal Medicine, Chi Mei Medical Center, Chiali, Tainan, Taiwan; 4 Department of Medical Research, Chi Mei Medical Center, Tainan, Taiwan; 5 Department of Information Management, Southern Taiwan University of Science and Technology, Tainan, Taiwan; 6 Cancer Center, Taipei Municipal Wanfang Hospital, Taipei Medical University, Taipei, Taiwan; 7 Department of Internal Medicine, National Taiwan University Hospital, Taipei, Taiwan

**Keywords:** Diabetes mellitus, SGLT2 inhibitors, Tuberculosis, Host-directed therapy

## Abstract

**Background:**

Sodium-glucose cotransporter 2 inhibitors (SGLT2i) are increasingly prescribed in diabetes mellitus (DM) patients, yet their impact on tuberculosis (TB) incidence remains unclear. This study analyzed TB risk in DM patients with and without SGLT2i use.

**Methods:**

We conducted a retrospective cohort study using data from the Taiwan National Health Insurance Research Database between 2017 to 2020. DM patients treated with or without SGLT2i were identified, and propensity score (PS) matching was performed to balance baseline characteristics, including age, sex, comorbidities, and Diabetes Complications Severity Index (DCSI) between these two groups. TB incidence was assessed until December 31, 2021, and compared between groups. To consider death as a competing risk, a Cox regression model with the Fine and Gray approach was used to estimate the incidence of TB. Additionally, sensitivity analyses were conducted by excluding patients with concomitant antidiabetic medications and by redefining the exposure period based on the timing of SGLT2i initiation.

**Results:**

After PS matching, 76,159 SGLT2i users and 152,318 non-users were included. The majority of enrolled patients were aged over 50 years (63.96%), with males predominant (61.92%). Over an average follow-up of 3.08 years, TB incidence was lower in SGLT2i users than in non-users (0.10% vs. 0.17%, SMD = 0.0192). The time to TB diagnosis was longer in SGLT2i users compared to non-users (2.56 ± 1.13 vs. 2.27 ± 0.02, SMD = -0.2853). Cox regression showed a reduced TB risk in SGLT2i users, with an adjusted hazard ratio of 0.43 (95% CI: 0.33–0.56). The protective effect persisted across age, gender and concomitant use of other antidiabetic drugs. A dose–response relationship was observed, where patients receiving higher cumulated defined daily doses of SGLT2i exhibited progressively lower TB risk. Finally, sensitivity analyses reinforced the robustness of the findings and suggested a temporal association between SGLT2i exposure and a reduced risk of TB occurrence.

**Conclusions:**

This study provides novel evidence that SGLT2i use is associated with a lower TB risk in DM patients, suggesting a potential host-directed therapeutic role warranting further investigation.

**Supplementary Information:**

The online version contains supplementary material available at 10.1186/s12916-025-04460-w.

## Background

Tuberculosis (TB) remains one of the most prevalent and deadly infectious diseases and a leading cause of death worldwide, with 10.8 million new cases and nearly 1.25 million deaths reported in 2023, despite effective strategies and public health awareness, according to the World Health Organization Global Tuberculosis Report 2024 [1]. Notably, TB incidence is higher among high risk populations, such as diabetes mellitus (DM) patients [2], post gastrectomy patients [3], human immunodeficiency virus (HIV)-infected patients [4] and immunocompromised patients [5].

Among the above health conditions, DM is rapidly increasing as an epidemic disease [6, 7] and has a two-to four-fold higher risk of active TB [8–10]. Notably, the incidence of active pulmonary tuberculosis is about 4% in DM patients, with the risk increasing further in those with poor glycemic control [11, 12]. By contrast, the prevalence of diabetes mellitus is 15% to 40% in pulmonary tuberculosis patients [13, 14]. Given the large population of DM patient [15], the associated immune dysfunction involving macrophages, Th1, and Th17 responses in controlling and clearing *Mycobacterium tuberculosis* (Mtb) [16–18], along with the higher treatment costs, longer hospital stays [19], and increased mortality observed in DM patients with TB [20, 21], reducing the risk of TB in this population is a critical public health priority.

Since dysregulated immunity in DM contributes to increased susceptibility to TB [18], selecting antidiabetic medications that help restore immune function may play a critical role in mitigating this risk. Among the treatments for type 2 DM, metformin reportedly has a protective effect against TB incidence [22], whereas dipeptidyl peptidase-4 (DPP-4) inhibitors have a neutral role in mycobacterial pulmonary infection [23]. Due to its potential anti-TB properties, metformin has been proposed as an adjunctive therapy for TB [24]. However, its adverse effects, including the risk of lactic acidosis in patients with renal impairment and gastrointestinal disturbances, limit its widespread use in DM patients. Recently, sodium-glucose cotransporter 2 inhibitors (SGLT2i) have been increasingly prescribed for their superior glycemic control and favorable safety profiles. Beyond their antidiabetic effects, SGLT2i exhibit pleiotropic effects, including reductions in the risk of sepsis and pneumonia [25]. Based on these observations, we tried to explore the potential association between SGLT2i use and TB incidence. However, to date, no preclinical or clinical studies have investigated the association between SGLT2i use and TB incidence in a DM population. To address this knowledge gap, we conducted a nationwide retrospective cohort study using the National Insurance Research Database. Our aim was to evaluate whether SGLT2i use was associated with a reduced risk of TB among patients with type 2 DM.

## Methods

### Study design and data sources

In this retrospective cohort study, we identified DM patients treated with or without SGLT2i between January 1, 2017 and December 31, 2020 using the Taiwan National Health Insurance Research Database (NHIRD), which covers > 99% of the nation’s residents and includes information on participants’ disease and medical treatment histories [26]. The index date was defined as one year after the initial diagnosis of DM, to create a standardized baseline window for covariates assessment and allow sufficient time for initial treatment patterns and comorbidity development. Exposure classification was based on SGLT2i use after this index date, which allowed consistent alignment between exposure and follow-up time and helped minimize the risk of immortal time bias. The follow-up period continued until December 31, 2021, to assess the occurrence of TB, death or loss to follow-up among DM patients with and without SGLT2i. Death was identified using Taiwan's cause-of-death registry, which is linked to the NHIRD.

### Study subjects

The definition of DM was based on the ICD-10-CM diagnosis codes (E08-E13), requiring at least one inpatient admission or three outpatient diagnoses within a 1-year period. The study focused on estimating the risk of TB among DM patients using SGLT2i, designating DM patients with SGLT2i as cases and those without as controls. SGLT2i use was defined by the ATC code, A10BK. To reduce selection bias and address potential confounding factors, propensity score (PS) matching was used for each case, with two controls selected from DM patients not using SGLT2i and matched based on age, gender, comorbidities, and the Diabetes Complications Severity Index (DCSI) score. These matching variables were chosen based on their established associations with both SGLT2i prescribing patterns and tuberculosis risk [27, 28]. The propensity score matching approach was based on the multivariable logistic regression model in an SAS matching macro, “%OneToManyMTCH,” which followed the use of a greedy algorithm to select cases and controls [29].

The exclusion criteria for this study included DM patients aged < 20 years and those who had only used SGLT2i before the DM diagnosis date, had TB prior to index date, had non-type 1 DM or non-type 2 DM, follow-up time of < 1 year, or < 30 days of SGLT2i use.

### Measurements and outcome

Baseline information among DM patients included age, gender, and comorbidities. All comorbidities were identified using ICD-10-CM codes extracted from 1-year inpatient and outpatient records after the date of DM diagnosis. The comorbidities were hyperlipidemia (ICD-10-CM: E78), hypertension (HTN; ICD-10-CM: I10-I13, I15), cardiovascular disease (CVD; ICD-10-CM: G45-G46, I60-I69, H34.0), chronic obstructive pulmonary disease (COPD; ICD-10-CM: J41-J44), asthma (ICD-10-CM: J45), and chronic kidney disease (CKD; ICD-10-CM: N18). The Charlson Comorbidity Index (CCI) score [30, 31] was calculated using diagnostic records from the one-year period prior to the diagnosis of DM to reflect baseline comorbidity status. The DCSI score [32] was assessed during the baseline period between the date of DM diagnosis and the index date to define early diabetes-related complications prior to SGLT2i exposure.

Additionally, defined daily doses (DDDs), following recommendations from the World Health Organization Collaborating Centre for Drug Statistics Methodology, were used to quantify drug use [33]. To assess the risk of increased TB incidence, cumulative DDDs (cDDDs), indicating the total exposed dosage of SGLT2i in DM patients, were calculated as the sum of dispensed DDDs of SGLT2i during the follow-up period. Given the absence of clinically established cDDDs thresholds, SGLT2i use was stratified into three levels based on the quantile distribution of cDDDs: < 68, 68–260, and > 260. This data-driven stratification used exploratory evaluation of potential dose–response relationships, and it was better suited to the highly skewed nature of the cDDDs distribution in the study population (Additional file 1: Figure S1).

Considering potential confounding from diabetes pharmacotherapy, baseline use of metformin, sulfonylureas (SU), DPP-4 inhibitors, and insulin was also included in this study during the follow-up periods. These medications were identified using ATC codes as follows: metformin (A10BA02, A10BD02, A10BD05, A10BD07, A10BD08, A10BD10, A10BD11, A10BD13, A10BD14, A10BD15, A10BD20), SU (A10BB12, A10BD02, A10BB01, A10BB07, A10BB09), DPP-4 inhibitors (A10BH01, A10BD07, A10BD24, A10BH02, A10BD08, A10BH03, A10BD10, A10BD21, A10BH05, A10BD11, A10BD19), and insulin (A10AB01, A10AB04, A10AB05, A10AB06, A10AC01, A10AE04, A10AE05, A10AE06, A10AD01, A10AD04, A10AD05, A10AD06, A10AE54). These variables were treated as covariates in the multivariable Cox regression models to control for differences in background treatment.

The primary outcome of the study was TB, defined based on the ICD-10-CM diagnosis codes (A15.0, A15.4-A15.9, A17-A19), requiring at least one inpatient admission or two outpatient diagnoses within a 6-month period. To prevent misclassification bias, study subjects were limited to patients with a TB diagnosis code who also received anti-TB prescriptions. Anti-TB prescriptions were identified using ATC codes, covering drugs such as isoniazid (ATC codes: J04AC01, J04AC51, J04AM02, J04AM03, J04AM05, J04AM06, J04AM07), rifampin (ATC codes: J04AB02, J04AC51, J04AM02, J04AM05, J04AM06, J04AM07), pyrazinamide (ATC codes: J04AK01, J04AM05, J04AM06), and ethambutol (ATC codes: J04AK02, J04AM03, J04AM06, J04AM07).

### Statistical analysis

Baseline characteristics between DM patients treated with SGLT2i and those without were compared using standardized mean differences (SMDs) to assess covariate balance. An SMD of less than 0.1 was considered to indicate adequate covariate balance [34]. The trends in incidence of TB between DM patients taking SGLT2i and those without were plotted with the Kaplan–Meier method, and differences were compared using the log-rank test. The risk of TB was presented as hazard ratios (HRs) with 95% confidence intervals (95% CIs) and calculated using the Cox proportional hazard model after adjusting for other confounding variables. As death may affect the occurrence of TB, we performed a competing risk analysis to estimate the TB incidence using the Cox regression model with the Fine and Gray approach. The proportional hazards assumption was assessed using Schoenfeld residuals. The global test showed no significant violation after matching (*p* = 0.2893), confirming the validity of the Cox model. The presentation of results included stratified analyses to estimate differences across groups. Additionally, subgroup analyses were conducted stratifying DM patients by cDDDs of SGLT2i, age, gender, and concomitant use of other antidiabetic drugs.

To address potential bias, we conducted several sensitivity analyses. First, we excluded patients who were diagnosed with TB within 30 days after the index date, based on the rationale that early TB diagnoses may reflect pre-existing, undetected infections rather than new-onset TB related to SGLT2i exposure. Second, to reduce confounding from concomitant treatments, we excluded patients in the SGLT2i group who received other antidiabetic medications during the follow-up period, allowing for a more specific evaluation of the independent effect of SGLT2i. Third, to evaluate the influence of exposure timing on outcome, the index date was redefined based on the actual initiation of SGLT2i therapy to better capture the relevant risk period. We then conducted a separate analysis for early initiation, defined as SGLT2i use within the first year after DM diagnosis, to assess the temporal relationship between exposure and TB incidence.

All statistical analyses were performed in SAS 9.4 for Windows (SAS Institute, Inc., Cary, NC, USA). Kaplan–Meier curves were plotted in STATA version 12 (Stata Corp., College Station, TX, USA) due to its flexibility in survival curve visualization. Statistical significance was set at 0.05 (two-tailed).

## Results

### Enrollment and clinical characteristics of patients

**Fig. 1:**
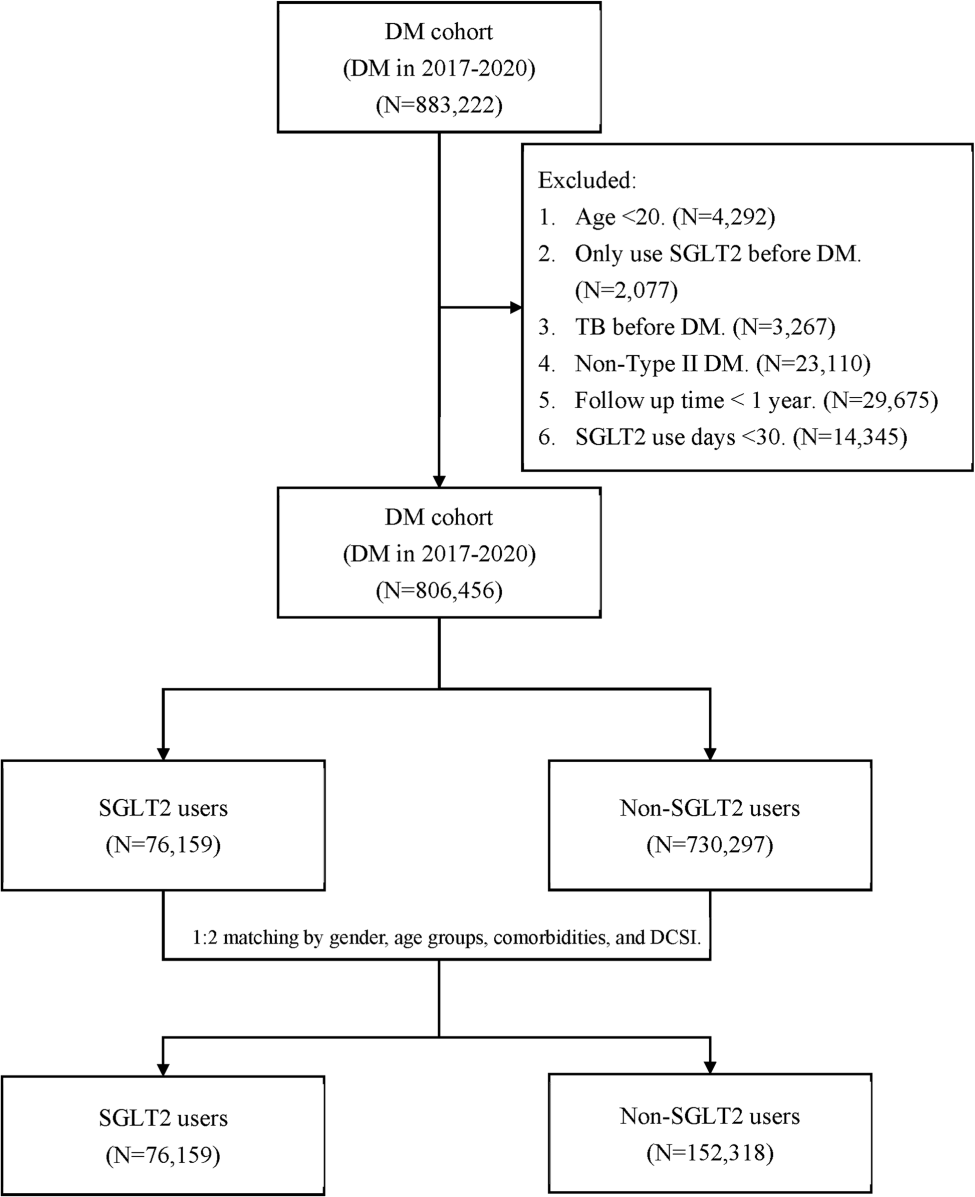
Flowchart of case selection and propensity score matching for diabetes mellitus (DM) patients treated with or without sodium-glucose cotransporter 2 (SGLT2) inhibitors

The enrollment details of the patients are illustrated in Fig. 1. The dataset for analysis comprised 883,222 patients diagnosed with DM between 2017 and 2020. Following the application of exclusion criteria, 76,766 patients were excluded from the study. Consequently, a total of 806,456 patients, consisting of 76,159 SGLT2i users and 730,297 non-SGLT2i users, were included in the final analysis.

Table 1 provides a summary of the clinical characteristics of the enrolled patients. The majority of enrolled patients were aged over 50 years (78.61%), with males slightly predominant (54.23%). In terms of DM classification, the vast majority (98.54%) were diagnosed with type 2 DM. In comparing SGLT2i users to non-SGLT2i users, it was observed that SGLT2i users were generally younger and had a higher percentage of males (61.90% vs. 53.43%, SMD = 0.2583). Additionally, SGLT2i users exhibited fewer comorbidities including HTN, CVD, COPD, asthma, and CKD. Regarding DM status and complications, SGLT2i users had higher rates of insulin use (33.56% vs. 20.15%, SMD = 0.3062), metformin use (95.53% vs. 68.47%, SMD = 0.7526), SU use (46.97% vs. 22.25%, SMD = 0.5382), and DPP-4 inhibitors use (35.96% vs. 19.63%, SMD = 0.3707), as well as a higher mean DCSI score (0.75 ± 1.06 vs. 0.65 ± 1.05, SMD = 0.1009).

**Table 1:** Baseline demographics of enrolled DM patients

	Before matching				After matching			
	Total DM patients (*N* = 806,456)	SGLT2i users (*N* = 76,159)	Non-SGLT2i users (*N* = 730,297)	SMD	Total DM patients (*N* = 228,477)	SGLT2i users (*N* = 76,159)	Non-SGLT2i users (*N* = 152,318)	SMD
Gender								
Male	437,304(54.23)	47,142(61.9)	390,162(53.43)	0.2583	141,467(61.92)	47,142(61.9)	94,325(61.93)	0.0095
Female	369,152(45.77)	29,017(38.1)	340,135(46.57)		87,010(38.08)	29,017(38.1)	57,993(38.07)	
Age group in index date								
20–39	56,082(6.95)	10,214(13.41)	45,868(6.28)	0.4630	30,676(13.43)	10,214(13.41)	20,462(13.43)	< 0.0001
40–49	116,441(14.44)	17,231(22.63)	99,210(13.58)		51,664(22.61)	17,231(22.63)	34,433(22.61)	
50–59	206,656(25.63)	21,561(28.31)	185,095(25.35)		64,695(28.32)	21,561(28.31)	43,134(28.32)	
60–69	235,724(29.23)	17,924(23.53)	217,800(29.82)		53,764(23.53)	17,924(23.53)	35,840(23.53)	
> = 70	191,553(23.75)	9229(12.12)	182,324(24.97)		27,678(12.11)	9229(12.12)	18,449(12.11)	
Comorbidity								
Hyperlipidemia	430,749(53.41)	46,096(60.53)	384,653(52.67)	−0.1590	138,253(60.51)	46,096(60.53)	92,157(60.5)	0.0005
HTN	442,303(54.85)	40,781(53.55)	401,522(54.98)	−0.0288	122,267(53.51)	40,781(53.55)	81,486(53.5)	0.0010
CVD	69,956(8.67)	4945(6.49)	65,011(8.9)	−0.0905	14,816(6.48)	9871(12.96)	4945(3.25)	0.0005
COPD	29,463(3.65)	1870(2.46)	27,593(3.78)	−0.0762	5568(2.44)	1870(2.46)	3698(2.43)	0.0018
Asthma	23,634(2.93)	1970(2.59)	21,664(2.97)	−0.0231	5869(2.57)	1970(2.59)	3899(2.56)	0.0017
CKD	49,496(6.14)	4053(5.32)	45,443(6.22)	−0.0386	12,144(5.32)	4053(5.32)	8091(5.31)	0.0004
DM type								
Type I	11,808(1.46)	1509(1.98)	10,299(1.41)	0.2411	4455(1.95)	1509(1.98)	2946(1.93)	−0.0007
Type II	794,648(98.54)	74,650(98.02)	719,998(98.59)		224,022(98.05)	74,650(98.02)	149,372(98.07)	
Other antidiabetic drug								
Insulin	172,706(21.42)	25,562(33.56)	147,144(20.15)	0.3062	57,618(25.22)	25,562(33.56)	32,056(21.05)	0.2838
Metformin	572,767(71.02)	72,754(95.53)	500,013(68.47)	0.7526	183,276(80.22)	72,754(95.53)	110,522(72.56)	0.6606
SU	198,229(24.58)	35,770(46.97)	162,459(22.25)	0.5382	73,549(32.19)	35,770(46.97)	37,779(24.8)	0.4749
DPP-4 inhibitors	170,758(21.17)	27,387(35.96)	143,371(19.63)	0.3707	58,223(25.48)	27,387(35.96)	30,836(20.24)	0.3551
CCI score	0.58 ± 1.18	0.41 ± 0.96	0.59 ± 1.20	−0.1633	0.46 ± 1.05	0.41 ± 0.96	0.49 ± 1.09	−0.0731
CCI group								
0	559,834(69.42)	57,562(75.58)	502,272(68.78)	0.1844	168,671(73.82)	57,562(75.58)	111,109(72.95)	0.1082
1–2	199,016(24.68)	15,862(20.83)	183,154(25.08)		49,980(21.88)	15,862(20.83)	34,118(22.4)	
≧3	47,606(5.9)	2735(3.59)	44,871(6.14)		9826(4.3)	2735(3.59)	7091(4.66)	
DCSI	0.66 ± 1.05	0.75 ± 1.06	0.65 ± 1.05	0.1009	0.75 ± 1.05	0.75 ± 1.06	0.75 ± 1.05	0.0020
DCSI group								
0	508,482(63.05)	465,357(611.03)	43,125(5.91)	0.1443	129,396(56.63)	43,125(56.62)	86,271(56.64)	< 0.0001
1	147,061(18.24)	130,423(171.25)	16,638(2.28)		49,961(21.87)	16,638(21.85)	33,323(21.88)	
≧2	150,913(18.71)	134,517(176.63)	16,396(2.25)		49,120(21.5)	16,396(21.53)	32,724(21.48)	
Diabetes complications								
Retinopathy	21,819(2.71)	3257(4.28)	18,562(2.54)	0.0957	9250(4.05)	3257(4.28)	5993(3.93)	0.0172
Nephropathy	108,894(13.5)	12,323(16.18)	96,571(13.22)	0.0836	36,144(15.82)	12,323(16.18)	23,821(15.64)	0.0148
Neuropathy	39,329(4.88)	4412(5.79)	34,917(4.78)	0.0452	13,826(6.05)	4412(5.79)	9414(6.18)	−0.0163
Cerebrovascular	51,139(6.34)	3780(4.96)	47,359(6.48)	−0.0655	11,583(5.07)	3780(4.96)	7803(5.12)	−0.0073
Cardiovascular	133,573(16.56)	15,540(20.4)	118,033(16.16)	0.1099	45,022(19.71)	15,540(20.4)	29,482(19.36)	0.0263
Peripheral Vascular	17,614(2.18)	1785(2.34)	15,829(2.17)	0.0119	6419(2.81)	1785(2.34)	4634(3.04)	−0.0432
Metabolic	12,324(1.53)	1551(2.04)	10,773(1.48)	0.0428	4896(2.14)	1551(2.04)	3345(2.2)	−0.0111
cDDD, mean ± SD	-	193.94 ± 181.94	-	-	-	193.94 ± 181.94	-	-
cDDD, median(Q1-Q3)	-	136(68–260)	-	-	-	136(68–260)	-	-
cDDD group								
< 68	-	18,975(24.91)	-	-	-	18,975(24.91)	-	-
68–260	-	38,231(50.2)	-		-	38,231(50.2)	-	
> 260	-	18,953(24.89)	-		-	18,953(24.89)	-	
Outcome								
Time to follow up	3.00 ± 1.17	3.30 ± 1.13	2.97 ± 1.16	0.2907	3.08 ± 1.17	3.30 ± 1.13	2.97 ± 1.17	0.2889
TB	1514(0.19)	75(0.1)	1439(0.2)	−0.0257	332(0.15)	75(0.1)	257(0.17)	0.0192
Time to TB	2.24 ± 0.91	2.56 ± 1.13	2.23 ± 0.90	0.3292	2.34 ± 0.98	2.56 ± 1.13	2.27 ± 0.92	−0.2853
Death	37,866(4.7)	1801(2.36)	36,065(4.94)	−0.1375	7022(3.07)	1801(2.36)	5221(3.43)	−0.0634

Data are numbers (percentage) or mean ± standard deviation

*Abbreviation: DM* diabetes mellitus, *SGLT2i* sodium-glucose cotransporter 2 inhibitors, *SMD* standardized mean difference, *HTN* hypertension, *CVD* cardiovascular disease, *COPD* chronic obstructive pulmonary disease, *CKD* chronic kidney disease, *SU* sulfonylureas, *DPP-4* dipeptidyl peptidase-4, *CCI* Charlson Comorbidity Index, *DCSI* Diabetes Complications Severity Index, *cDDD* cumulative defined daily dose, *TB* tuberculosis

### PS matched cohort

In the PS matched cohort, a matched cohort of 76,159 SGLT2i users and 152,318 non-SGLT2i users was meticulously assembled by balancing the age, gender, comorbidities, and DCSI (Fig. 1). As shown in Table 1, following the matching process, no statistical differences were observed in terms of age, gender, comorbidities, DCSI score, diabetes complications, or DM classification between the SGLT2i users and non-SGLT2i users (all SMD < 0.1). Considering DM status and complications, SGLT2i users exhibited a higher percentage of insulin use (33.56% vs. 21.05%, SMD = 0.2838), metformin use (95.53% vs. 72.56%, SMD = 0.6606), SU use (46.97% vs. 24.80%, SMD = 0.4749), and DPP-4 inhibitors use (35.96% vs. 20.24%, SMD = 0.3551).

### The risk of incident TB

Over an average follow-up period of three years **(**Table 1**)**, the incidence of newly diagnosed TB was lower in SGLT2i users than in non-users, both before PS matching (0.1% vs. 0.2%, SMD = −0.0257) and after PS matching (0.10% vs. 0.17%, SMD = 0.0192). Additionally, the time to newly diagnosed TB was longer in SGLT2i users than in non-users, both before PS matching (2.56 ± 1.13 vs. 2.23 ± 0.90 years, SMD = 0.3292) and after PS matching (2.56 ± 1.13 vs. 2.27 ± 0.92 years, SMD = −0.2853).

**Fig. 2:**
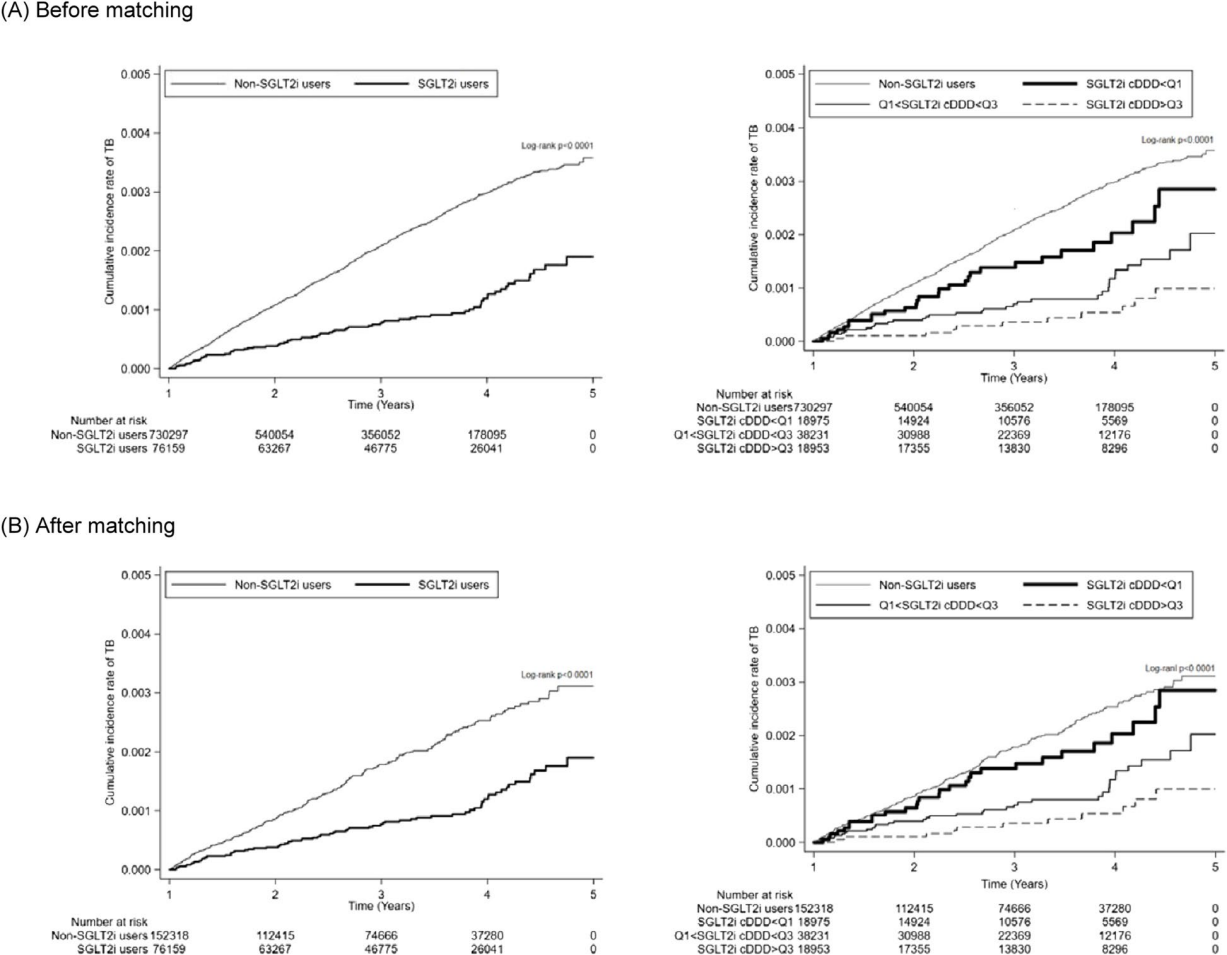
Kaplan–Meier survival curves depicting the cumulative incidence of tuberculosis (TB) in patients treated with or without sodium-glucose cotransporter 2 inhibitors (SGLT2i), stratified by cumulative defined daily doses (cDDDs), shown before (**a**) and after (**b**) propensity score matching

The Kaplan–Meier survival curves (Figs. 2a and b) also demonstrated a lower cumulative incidence of TB among SGLT2i users in both cohorts before and after PS matching (both *p* < 0.001).

### The risk of incident TB adjusted for confounding variables

Before covariate adjustment (Table 2), SGLT2i users exhibited protective crude HRs for incident TB of 0.44 (95% CI: 0.35–0.56) and 0.50 (95% CI: 0.39–0.65) compared to non-SGLT2i users before and after PS matching, respectively. After adjustments for covariates, including age, gender, other antidiabetic drug (including insulin use, metformin use, SU use, and DPP-4 inhibitors use), comorbidities, CCI group, and DCSI group, SGLT2i users continued to show a significantly lower incidence of TB, with adjusted HRs (AHR) of 0.49 (95% CI: 0.38–0.62) and 0.43 (95% CI: 0.33–0.56) before and after PS matching, respectively.

**Table 2:** The risk of TB in DM patients under competing risk analysis adjusted for confounding variables

	Before matching							After matching						
	Patients (*N* = 806,456)	TB (*N* = 1514)	Death (*N* = 37,489)	Crude HR (95% CI)	*p*-value	AHR (95% CI)	*p*-value	Patients (*N* = 228,477)	TB (*N* = 332)	Death (*N* = 6767)	Crude HR (95% CI)	*p*-value	AHR (95% CI)	*p*-value
SGLT2i	76,159	75(0.1)	1786(2.35)	0.44(0.35–0.56)	< 0.0001	0.49(0.38–0.62)	< 0.0001	76,159	75(0.1)	1786(2.35)	0.50(0.39–0.65)	< 0.0001	0.43(0.33–0.56)	< 0.0001
Non-SGLT2i	730,297	1439(0.2)	35,703(4.89)	Ref		Ref		152,318	257(0.17)	5181(3.4)	Ref		Ref	
Gender														
Male	437,304	1111(0.25)	22,361(5.11)	2.33(2.08–2.61)	< 0.0001	2.45(2.18–2.76)	< 0.0001	141,467	257(0.18)	4900(3.46)	-		-	
Female	369,152	403(0.11)	15,128(4.1)	Ref		Ref		87,010	75(0.09)	2067(2.38)	-		-	
Age group														
20–39	56,082	40(0.07)	430(0.77)	Ref		Ref		30,676	14(0.05)	200(0.65)	-		-	
40–49	116,441	122(0.1)	1767(1.52)	1.47(1.03–2.10)	0.0348	1.57(1.10–2.24)	0.0140	51,664	45(0.09)	751(1.45)	-		-	
50–59	206,656	250(0.12)	4439(2.15)	1.66(1.19–2.32)	0.0028	1.91(1.36–2.67)	0.0002	64,695	79(0.12)	1322(2.04)	-		-	
60–69	235,724	410(0.17)	7694(3.26)	2.41(1.74–3.33)	< 0.0001	2.74(1.98–3.81)	< 0.0001	53,764	103(0.19)	1754(3.26)	-		-	
> = 70	191,553	692(0.36)	23,159(12.09)	4.98(3.62–6.85)	< 0.0001	4.54(3.26–6.31)	< 0.0001	27,678	91(0.33)	2940(10.62)	-		-	
Other antidiabetic drug														
Insulin	172,706	825(0.48)	25,037(14.5)	3.92(3.54–4.33)	< 0.0001	3.63(3.23–4.09)	< 0.0001	57,618	191(0.33)	4975(8.63)	3.11(2.48–3.91)	< 0.0001	3.41(2.67–4.35)	< 0.0001
Metformin	572,767	990(0.17)	21,695(3.79)	0.72(0.65–0.80)	< 0.0001	0.77(0.68–0.86)	< 0.0001	183,276	254(0.14)	4863(2.65)	0.87(0.67–1.13)	0.2969	0.81(0.60–1.09)	0.1611
SU	198,229	458(0.23)	10,760(5.43)	1.14(1.02–1.27)	0.0209	1.07(0.95–1.21)	0.2617	73,549	134(0.18)	2598(3.53)	1.28(1.03–1.59)	0.0253	1.22(0.96–1.57)	0.1077
DPP-4	170,758	427(0.25)	13,830(8.1)	1.28(1.15–1.43)	< 0.0001	0.83(0.73–0.94)	0.0027	58,223	118(0.2)	2897(4.98)	1.21(0.96–1.52)	0.1034	1.02(0.80–1.29)	0.9055
Comorbidity														
Hyperlipidemia	430,749	569(0.13)	10,651(2.47)	0.54(0.48–0.60)	< 0.0001	0.76(0.68–0.85)	< 0.0001	138,253	150(0.11)	2592(1.87)	-		-	
HTN	442,303	891(0.2)	25,122(5.68)	1.17(1.05–1.29)	0.0032	0.91(0.82–1.01)	0.0889	122,267	179(0.15)	4475(3.66)	-		-	
CVD	69,956	237(0.34)	9437(13.49)	1.88(1.64–2.16)	< 0.0001	1.03(0.88–1.21)	0.7020	14,816	39(0.26)	1297(8.75)	-		-	
COPD	29,463	187(0.63)	5591(18.98)	3.52(3.02–4.11)	< 0.0001	1.51(1.27–1.80)	< 0.0001	5568	30(0.54)	718(12.9)	-		-	
Asthma	23,634	84(0.36)	1861(7.87)	1.90(1.52–2.37)	< 0.0001	1.34(1.06–1.69)	0.0136	5869	15(0.26)	293(4.99)	-		-	
CKD	49,496	204(0.41)	7383(14.92)	2.37(2.04–2.74)	< 0.0001	1.23(1.04–1.46)	0.0170	12,144	28(0.23)	1037(8.54)	-		-	
CCI group														
0	559,834	837(0.15)	13,751(2.46)	Ref		Ref		168,671	202(0.12)	3039(1.8)	Ref		Ref	
1–2	199,016	475(0.24)	13,674(6.87)	1.58(1.41–1.77)	< 0.0001	1.03(0.91–1.17)	0.6016	49,980	92(0.18)	2318(4.64)	1.02(0.78–1.34)	0.8669	1.06(0.81–1.38)	0.6813
≧3	47,606	202(0.42)	10,064(21.14)	2.81(2.41–3.28)	< 0.0001	1.07(0.89–1.28)	0.4919	9826	38(0.39)	1610(16.39)	1.66(1.12–2.47)	0.0124	1.38(0.93–2.04)	0.1117
DCSI group														
0	508,482	770(0.15)	14,256(2.8)	Ref		Ref		129,396	144(0.11)	2245(1.73)	-		-	
1	147,061	267(0.18)	5948(4.04)	1.19(1.03–1.36)	0.0160	0.92(0.80–1.06)	0.2534	49,961	69(0.14)	1357(2.72)	-		-	
≧2	150,913	477(0.32)	17,285(11.45)	2.01(1.79–2.25)	< 0.0001	0.96(0.83–1.11)	0.5623	49,120	119(0.24)	3365(6.85)	-		-	

Data are numbers (percentage) or hazard ratio (95% confidence interval)

*Abbreviation: TB* tuberculosis, *DM* diabetes mellitus, *HR* hazard ratio, *AHR* adjusted hazard ratio, *CI* confidence interval, *SGLT2i* sodium-glucose cotransporter 2 inhibitors, *SU* sulfonylureas, *DPP-4* dipeptidyl peptidase-4, *HTN* hypertension, *CVD* cardiovascular disease, *COPD* chronic obstructive pulmonary disease, *CKD* chronic kidney disease, *CCI* Charlson Comorbidity Index, *DCSI* Diabetes Complications Severity Index

### Subgroup analysis – different cDDDs of SGLT2i

Furthermore, we evaluated the effects of different SGLT2i doses on the incidence of TB (Table 3). After PS matching and adjustment for covariates, including age, gender, other antidiabetic drug, comorbidities, CCI group, and DCSI group, SGLT2i users with cDDDs > 260, and between 68 and 260, exhibited significantly lower AHRs for incident TB of 0.21 (95% CI: 0.12–0.39), and 0.42 (95% CI: 0.29–0.60), respectively, compared to non-SGLT2i users.

**Table 3:** The risk of TB in DM patients stratified by SGLT2i dose (cDDDs) under competing risk analysis adjusted for confounding variables

	Before matching							After matching						
	Patients (*N* = 806,456)	TB (*N* = 1514)	Death (*N* = 37,489)	Crude HR (95% CI)	*p*-value	AHR (95% CI)	*p*-value	Patients (*N* = 228,477)	TB (*N* = 332)	Death (*N* = 6767)	Crude HR (95% CI)	*p*-value	AHR (95% CI)	*p*-value
SGLT2i														
Non-SGLT2i	730,297	1439(0.2)	35,703(4.89)	Ref		Ref		152,318	257(0.17)	5181(3.4)	Ref		Ref	
< 68	18,975	29(0.15)	613(3.23)	0.73(0.51–1.05)	0.0915	0.77(0.53–1.12)	0.1686	18,975	29(0.15)	613(3.23)	0.84(0.57–1.23)	0.3659	0.70(0.47–1.04)	0.0761
68–260	38,231	35(0.09)	792(2.07)	0.42(0.30–0.59)	< 0.0001	0.48(0.34–0.67)	< 0.0001	38,231	35(0.09)	792(2.07)	0.49(0.34–0.70)	< 0.0001	0.42(0.29–0.60)	< 0.0001
> 260	18,953	11(0.06)	381(2.01)	0.23(0.13–0.42)	< 0.0001	0.26(0.14–0.46)	< 0.0001	18,953	11(0.06)	381(2.01)	0.25(0.14–0.45)	< 0.0001	0.21(0.12–0.39)	< 0.0001
Gender														
Male	437,304	1111(0.25)	22,361(5.11)	2.33(2.08–2.61)	< 0.0001	2.46(2.19–2.76)	< 0.0001	141,467	257(0.18)	4900(3.46)	-		-	
Female	369,152	403(0.11)	15,128(4.1)	Ref		Ref		87,010	75(0.09)	2067(2.38)	-		-	
Age group														
20–39	56,082	40(0.07)	430(0.77)	Ref		Ref		30,676	14(0.05)	200(0.65)	-		-	
40–49	116,441	122(0.1)	1767(1.52)	1.47(1.03–2.10)	0.0348	1.57(1.10–2.24)	0.0135	51,664	45(0.09)	751(1.45)	-		-	
50–59	206,656	250(0.12)	4439(2.15)	1.66(1.19–2.32)	0.0028	1.91(1.37–2.67)	0.0002	64,695	79(0.12)	1322(2.04)	-		-	
60–69	235,724	410(0.17)	7694(3.26)	2.41(1.74–3.33)	< 0.0001	2.75(1.98–3.82)	< 0.0001	53,764	103(0.19)	1754(3.26)	-		-	
> = 70	191,553	692(0.36)	23,159(12.09)	4.98(3.62–6.85)	< 0.0001	4.54(3.26–6.32)	< 0.0001	27,678	91(0.33)	2940(10.62)	-		-	
Other antidiabetic drug														
Insulin	172,706	825(0.48)	25,037(14.5)	3.92(3.54–4.33)	< 0.0001	3.63(3.23–4.09)	< 0.0001	57,618	191(0.33)	4975(8.63)	3.11(2.48–3.91)	< 0.0001	3.42(2.67–4.37)	< 0.0001
Metformin	572,767	990(0.17)	21,695(3.79)	0.72(0.65–0.80)	< 0.0001	0.77(0.68–0.86)	< 0.0001	183,276	254(0.14)	4863(2.65)	0.87(0.67–1.13)	0.2969	0.81(0.60–1.09)	0.1680
SU	198,229	458(0.23)	10,760(5.43)	1.14(1.02–1.27)	0.0209	1.07(0.95–1.21)	0.2628	73,549	134(0.18)	2598(3.53)	1.28(1.03–1.59)	0.0253	1.22(0.95–1.56)	0.1133
DPP-4	170,758	427(0.25)	13,830(8.1)	1.28(1.15–1.43)	< 0.0001	0.83(0.73–0.93)	0.0021	58,223	118(0.2)	2897(4.98)	1.21(0.96–1.52)	0.1034	1.00(0.78–1.27)	0.9705
Comorbidity														
Hyperlipidemia	430,749	569(0.13)	10,651(2.47)	0.54(0.48–0.60)	< 0.0001	0.76(0.68–0.85)	< 0.0001	138,253	150(0.11)	2592(1.87)	-		-	
HTN	442,303	891(0.2)	25,122(5.68)	1.17(1.05–1.29)	0.0032	0.91(0.82–1.02)	0.0927	122,267	179(0.15)	4475(3.66)	-		-	
CVD	69,956	237(0.34)	9437(13.49)	1.88(1.64–2.16)	< 0.0001	1.03(0.88–1.21)	0.7156	14,816	39(0.26)	1297(8.75)	-		-	
COPD	29,463	187(0.63)	5591(18.98)	3.52(3.02–4.11)	< 0.0001	1.51(1.27–1.80)	< 0.0001	5568	30(0.54)	718(12.9)	-		-	
Asthma	23,634	84(0.36)	1861(7.87)	1.90(1.52–2.37)	< 0.0001	1.34(1.06–1.69)	0.0137	5869	15(0.26)	293(4.99)	-		-	
CKD	49,496	204(0.41)	7383(14.92)	2.37(2.04–2.74)	< 0.0001	1.23(1.04–1.46)	0.0184	12,144	28(0.23)	1037(8.54)	-		-	
CCI group														
0	559,834	837(0.15)	13,751(2.46)	Ref		Ref		168,671	202(0.12)	3039(1.8)	Ref		Ref	
1–2	199,016	475(0.24)	13,674(6.87)	1.58(1.41–1.77)	< 0.0001	1.04(0.91–1.17)	0.5845	49,980	92(0.18)	2318(4.64)	1.02(0.78–1.34)	0.8669	1.06(0.82–1.38)	0.6460
≧3	47,606	202(0.42)	10,064(21.14)	2.81(2.41–3.28)	< 0.0001	1.07(0.89–1.28)	0.4845	9826	38(0.39)	1610(16.39)	1.66(1.12–2.47)	0.0124	1.38(0.93–2.04)	0.1086
DCSI group														
0	508,482	770(0.15)	14,256(2.8)	Ref		Ref		129,396	144(0.11)	2245(1.73)	-		-	
1	147,061	267(0.18)	5948(4.04)	1.19(1.03–1.36)	0.0160	0.92(0.80–1.07)	0.2688	49,961	69(0.14)	1357(2.72)	-		-	
≧2	150,913	477(0.32)	17,285(11.45)	2.01(1.79–2.25)	< 0.0001	0.96(0.83–1.11)	0.5987	49,120	119(0.24)	3365(6.85)	-		-	

Data are numbers (percentage) or hazard ratio (95% confidence interval)

*Abbreviation: TB* tuberculosis, *DM* diabetes mellitus, *cDDDs* cumulative defined daily doses, *HR* hazard ratio, *AHR* adjusted hazard ratio, *CI* confidence interval, *SGLT2i* sodium-glucose cotransporter 2 inhibitors, *SU* sulfonylureas, *DPP-4* dipeptidyl peptidase-4, *HTN* hypertension, *CVD* cardiovascular disease, *COPD* chronic obstructive pulmonary disease, *CKD* chronic kidney disease, *CCI* Charlson Comorbidity Index, *DCSI* Diabetes Complications Severity Index

The Kaplan–Meier survival curves (Figs. 2a and b) also demonstrated a consistently lower cumulative incidence of TB among SGLT2i users with higher cDDDs compared to non-SGLT2i users, both before and after PS matching (*p* < 0.001).

### Subgroup analysis – stratified by age, gender and concomitant use of other antidiabetic drugs

In the subgroup analysis stratified by age, gender and concomitant use of other antidiabetic drugs (Additional file 1: Table S1), the AHRs for incident TB remained significantly lower in SGLT2i users than in non-users across all subgroups. The protective association was observed in both males (AHR 0.41, 95% CI: 0.30–0.56) and females (AHR 0.57, 95% CI: 0.34–0.95); in patients < 65 years (AHR 0.43, 95% CI: 0.30–0.61) and ≥ 65 years (AHR 0.46, 95% CI: 0.31–0.68); and among those using other antidiabetic agents, including insulin (AHR 0.50, 95% CI: 0.37–0.48), metformin (AHR 0.45, 95% CI: 0.34–0.59), SU (AHR 0.47, 95% CI: 0.33–0.67), and DPP-4 inhibitors (AHR 0.59, 95% CI: 0.41–0.86), even after propensity score matching and adjustment for covariates.

### Sensitivity analysis

Considering the rationale that early TB diagnoses may reflect pre-existing disease rather than SGLT2i-associated risk, a sensitivity analysis was presented excluding patients who were diagnosed with TB within 30 days after the index date (Additional file 1: Table S2). A PS-matched cohort of 76,156 SGLT2i users and 152,312 non-SGLT2i users was established, and SGLT2i users had significantly lower TB risk than non SGLT2i users (AHR: 0.44; 95% CI: 0.33–0.57). The other sensitivity analysis was performed to address potential confounding from concomitant treatments (Additional file 1: Table S3). After excluding patients in the SGLT2i group who also received other antidiabetic medications, SGLT2i users show 0.37-fold risk of TB (95% CI: 0.21–0.65) compared with non-SGLT2i users. Additionally, to address potential time-related biases, a sensitivity analysis of using SGLT2i initiation date as the index date (Additional file 1: Table S4). The results indicated a significantly lower TB risk in SGLT2i users than in non-users (AHR: 0.75; 95% CI: 0.57–0.99). When restricting exposure to early initiation (within the first year after DM diagnosis), SGLT2i users consistently exhibited a significantly lower risk of TB compared with non-users.

## Discussion

Using a large cohort of DM patients identified from the Taiwan NHIRD, this study is the first to report an association between SGLT2i use and a lower incidence of subsequent TB development in this population. This association remained consistent across different age groups, sexes, and concomitant antidiabetic therapies, suggesting a potential class effect of SGLT2i. Moreover, the cDDD analysis indicated a possible dose–response relationship, with lower TB incidence observed among patients receiving higher cumulative doses of SGLT2i. Finally, several sensitivity analyses were performed to minimize immortal time bias and better assess the temporal sequence of exposure and outcome. Collectively, these findings support a robust association between SGLT2i use and reduced TB risk, although causality cannot be inferred from this observational study.

The pathogenesis underlying the association between DM and TB is believed to be primarily related to dysfunctional host immunity with chronic inflammatory milieu. This dysfunction involves reduced Th1 and Th17 responses in LTBI patients with DM and hyperreactive but ineffective Th1 and Th17 responses in active TB patients with DM, leading to impaired Mtb killing and clearance [35, 36]. Based on these observations, host-directed therapy aimed at restoring immune dysfunction both to reduce the susceptibility of DM patients to TB is an appealing approach that warrants further research.

Metformin has been reported as an effective adjunctive therapy against TB [24]. Its protective effects extend beyond glycemic control and are believed to be related to enhancing macrophage activity and promoting inflammatory killing of Mtb [37]. In our analysis (Table 2), metformin users only showed a numerically lower, but not statistically significant, risk of TB compared with non-users (AHR: 0.81; 95% CI: 0.60–1.09). This finding underscores the need to explore additional therapeutic agents with potential protective effects in DM patients at risk of TB.

Our findings support SGLT2i’s potential as adjunctive agents for TB prevention. While the mechanisms remain unclear, several hypotheses can be proposed beyond its glycemic control effects including SGLT2i exhibit antioxidant properties and reduce systemic inflammation [38]. Additionally, emerging evidence suggests that SGLT2i may modulate the gut microbiota, particularly by reducing *Phascolarctobacterium*, a propionate-producing genus enriched in TB patients and associated with impaired macrophage function and dysregulated Th1 and Th17 responses [39–41]. This hypothesis points to a potential gut–lung axis mechanism through which SGLT2i could help reduce TB risk, warranting further mechanistic investigation.

The strengths of the present study included the large case number and stringent drug coding in a national insurance database. In addition, the findings have been validated by several subgroup analyses and sensitivity tests. Despite these significant findings, several limitations should be acknowledged. First, as a retrospective study using NHIRD data, confounding by indication remains a concern and may not be fully eliminated despite PS matching, as SGLT2i could have been preferentially prescribed to specific DM patients. In our dataset, SGLT2i users were generally younger, had fewer comorbidities, higher rates of concomitant use of other antidiabetic drugs (including insulin, metformin, sulfonylureas, and DPP-4 inhibitors), and a higher mean DCSI score. Furthermore, due to the inherent limitations of administrative claims data, certain clinical and social variables, such as body mass index, glycated hemoglobin (HbA1c), socioeconomic status, and medication adherence, were not available. In particular, HbA1c, an important marker of glycemic control that influences TB susceptibility, could not be adjusted for in our analysis. Although DCSI was used as a surrogate for diabetes severity, residual confounding related to differences in baseline glycemic status may persist.

Second, the diagnoses of TB and comorbidities were based on ICD-10 coding. Although previous validation studies have reported moderate to high sensitivity and positive predictive values for the Taiwan NHIRD [26], some degree of misclassification may still be present. Third, we could not account for the LTBI status and receipt of TB preventive therapy among enrolled DM patients, which might have influenced the observed association. Fourth, to address potential confounding from concomitant antidiabetic treatments, a sensitivity analysis was performed excluding patients in the SGLT2i group who also received other antidiabetic medications. While this approach allowed for a more specific evaluation of the independent effect of SGLT2 inhibitors, it may have introduced selection bias by preferentially retaining patients with less severe DM, who are more likely to receive monotherapy and inherently have a lower risk of TB. Importantly, the persistence of a protective association in this lower-risk subgroup suggests that the observed benefit of SGLT2i may be robust and not solely attributable to differences in baseline diabetes severity. Finally, as this study was based on Taiwan NHIRD data, its generalizability to populations with different TB incidence rates, diabetes management practices, or genetic backgrounds remains uncertain.

## Conclusions

SGLT2i use was associated with a lower risk of TB development in DM patients. While this finding suggests a potential role for SGLT2i as a host-directed therapy against TB, it should be interpreted cautiously given the observational nature of the study. Further prospective studies and randomized controlled trials are needed to elucidate the underlying mechanisms, confirm this association, and assess whether SGLT2i could have a preventive role in high TB-risk populations.

## Supplementary Information

Additional file 1: Figure S1 and Tables S1–S4. Figure S1. The distribution of cumulative defined daily doses (cDDDs) of SGLT2i was highly skewed in the study population. Table S1. The risk of TB among SGLT2i users and non-SGLT2i users stratified by gender, age, and other antidiabetic drug groups after propensity matching. Table S2. Sensitivity analysis of excluding patients who were diagnosed with TB within 30 days after the index date. Table S3. Sensitivity analysis excluding patients receiving other antidiabetic medications during the follow up periods in the SGLT2i group. Table S4. Sensitivity analysis using SGLT2i initiation date as the index date.

## Data Availability

The datasets used and/or analyzed during the current study are available from the corresponding author on reasonable request.

## Abbreviations

AHR: Adjusted hazard ratios
CCI: Charlson Comorbidity Index
cDDDs: Cumulative defined daily doses
CIs: Confidence intervals
CKD: Chronic kidney disease
COPD: Chronic obstructive pulmonary disease
CVD: Cardiovascular disease
DCSI: Diabetes Complications Severity Index
DDDs: Defined daily doses
DM: Diabetes mellitus
DPP-4: Dipeptidyl peptidase-4
HbA1c: Glycated hemoglobin
HRs: Hazard ratios
HTN: Hypertension
LTBI: Latent tuberculosis infection
Mtb: *Mycobacterium tuberculosis*
NHIRD: National Health Insurance Research Database
PS: Propensity score
SGLT2i: Sodium-glucose cotransporter 2 inhibitors
SMD: Standardized mean differences
SU: Sulfonylureas
TB: Tuberculosis
