# Supplementary material for: Sodium-glucose transport protein 2 inhibitor use in diabetes and its association with tuberculosis incidence
Source: BMC Med. 2025 Nov 17;23:635. doi: 10.1186/s12916-025-04460-w (PMC12625201; doi:10.1186/s12916-025-04460-w)
Supplement: Supplementary file 1 — Additional file 1: Figure S1 and Tables S1–S4. Figure S1. The distribution of cumulative defined daily doses (cDDDs) of SGLT2i was highly skewed in the study population. Table S1. The risk of TB among SGLT2i users and non-SGLT2i users stratified by gender, age, and other antidiabetic drug groups after propensity matching. Table S2. Sensitivity analysis of excluding patients who were diagnosed with TB within 30 days after the index date. Table S3. Sensitivity analysis excluding patients receiving other antidiabetic medications during the follow up periods in the SGLT2i group. Table S4. Sensitivity analysis using SGLT2i initiation date as the index date. [file 12916_2025_4460_MOESM1_ESM.docx]

**Figure S1.** The distribution of cumulative defined daily doses (cDDDs) of SGLT2i was highly skewed in the study population.


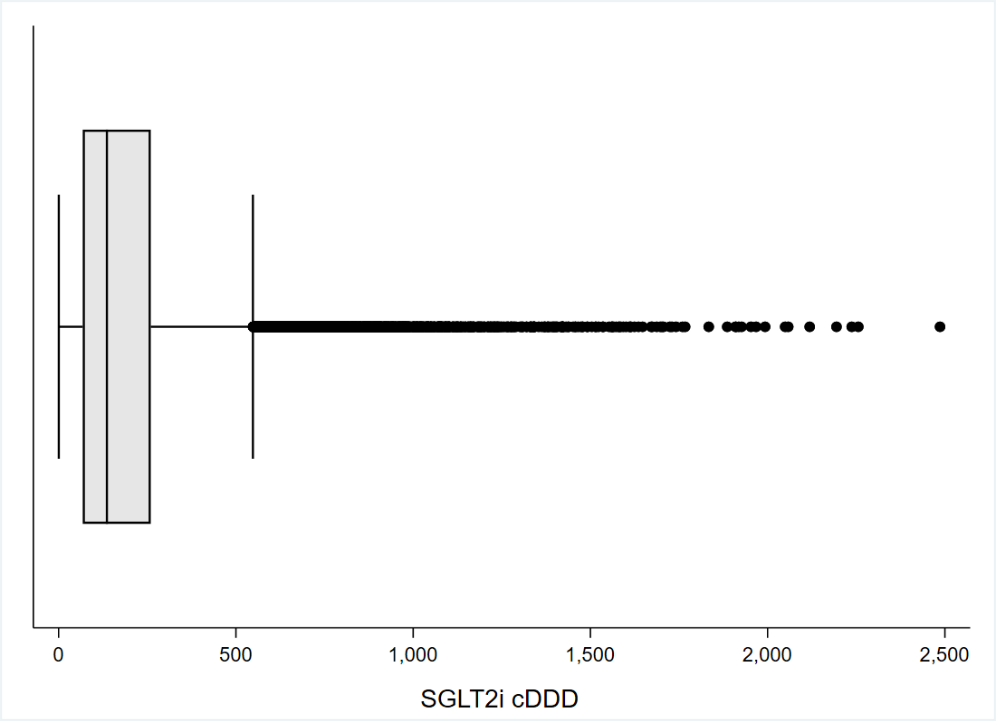


**Table S1.** The risk of TB among SGLT2i users and non-SGLT2i users stratified by gender, age, and other antidiabetic drug groups after propensity matching

| SGLT2i users vs. Non-SGLT2i users | SGLT2i users | | | Non-SGLT2i users | | |  |  |  |  |
| --- | --- | --- | --- | --- | --- | --- | --- | --- | --- | --- |
|  | Patients | TB | Death | Patients | TB | Death | Crude HR (95% CI) | p-value | AHR (95% CI) ^a^ | p-value |
| Overall | 76159 | 75(0.1) | 1786(2.35) | 152318 | 257(0.17) | 5181(3.40) | 0.50(0.39-0.65) | <0.0001 | 0.43(0.33-0.56) | <0.0001 |
| Stratified |  |  |  |  |  |  |  |  |  |  |
| Gender |  |  |  |  |  |  |  |  |  |  |
| Male | 47142 | 55(0.12) | 1264(2.68) | 94325 | 202(0.21) | 3636(3.85) | 0.48(0.36-0.64) | <0.0001 | 0.41(0.30-0.56) | <0.0001 |
| Female | 29017 | 20(0.07) | 522(1.8) | 57993 | 55(0.09) | 1545(2.66) | 0.61(0.37-1.02) | 0.0615 | 0.57(0.34-0.95) | 0.0314 |
| Age group |  |  |  |  |  |  |  |  |  |  |
| <65 | 58838 | 44(0.07) | 816(1.39) | 116446 | 142(0.12) | 2277(1.96) | 0.53(0.38-0.74) | 0.0002 | 0.43(0.30-0.61) | <0.0001 |
| ≥65 | 17321 | 31(0.18) | 970(5.6) | 35872 | 115(0.32) | 2904(8.1) | 0.50(0.34-0.75) | 0.0007 | 0.46(0.31-0.68) | 0.0001 |
| Other antidiabetic drug |  |  |  |  |  |  |  |  |  |  |
| Insulin | 25562 | 56(0.22) | 1457(5.7) | 32056 | 135(0.42) | 3518(10.97) | 0.47(0.35-0.64) | <0.0001 | 0.50(0.37-0.68) | <0.0001 |
| Metformin | 72754 | 72(0.1) | 1663(2.29) | 110522 | 182(0.16) | 3200(2.9) | 0.52(0.40-0.69) | <0.0001 | 0.45(0.34-0.59) | <0.0001 |
| SU | 35770 | 45(0.13) | 973(2.72) | 37779 | 89(0.24) | 1625(4.3) | 0.48(0.34-0.69) | <0.0001 | 0.47(0.33-0.67) | <0.0001 |
| DPP-4 inhibitors | 27387 | 43(0.16) | 1000(3.65) | 30836 | 75(0.24) | 1897(6.15) | 0.56(0.38-0.81) | 0.0021 | 0.59(0.41-0.86) | 0.0061 |

Data are numbers (percentage) or hazard ratio (95% confidence interval).

Abbreviation: TB, tuberculosis; HR, hazard ratio; AHR, adjusted hazard ratio; CI, confidence interval; SGLT2i, sodium-glucose cotransporter 2 inhibitors; SU, sulfonylureas; DPP-4, dipeptidyl peptidase-4;

^a^ adjusted by CCI groups and other antidiabetic drug use (including Insulin, Metformin, SU and DPP-4).

**Table S2.** Sensitivity analysis of excluding patients who were diagnosed with TB within 30 days after the index date

|  | Before matching | | | | | After matching | | | | |
| --- | --- | --- | --- | --- | --- | --- | --- | --- | --- | --- |
|  | Patients  (N=806378) | TB  (N=1436) | Death  (N=37489) | AHR  (95% CI) | p-value | Patients  (N=228468) | TB  (N=307) | Death  (N=6828) | AHR  (95% CI) | p-value |
| SGLT2i | 76156 | 72(0.09) | 1786(2.35) | 0.49(0.39-0.63) | <0.0001 | 76156 | 72(0.09) | 1786(2.35) | 0.44(0.33-0.57) | <0.0001 |
| Non-SGLT2i | 730222 | 1364(0.19) | 35703(4.89) | Ref. |  | 152312 | 235(0.15) | 5042(3.31) | Ref. |  |
| Gender |  |  |  |  |  |  |  |  |  |  |
| Male | 437250 | 1057(0.24) | 22361(5.11) | 2.49(2.21-2.81) | <0.0001 | 141432 | 251(0.18) | 4827(3.41) | - |  |
| Female | 369128 | 379(0.1) | 15128(4.1) | Ref. |  | 87036 | 56(0.06) | 2001(2.3) | - |  |
| Age group |  |  |  |  |  |  |  |  |  |  |
| 20-39 | 56078 | 36(0.06) | 430(0.77) | Ref. |  | 30697 | 15(0.05) | 222(0.72) | - |  |
| 40-49 | 116433 | 114(0.1) | 1767(1.52) | 1.62(1.12-2.36) | 0.0114 | 51652 | 40(0.08) | 712(1.38) | - |  |
| 50-59 | 206642 | 236(0.11) | 4439(2.15) | 2.00(1.40-2.84) | 0.0001 | 64658 | 68(0.11) | 1257(1.94) | - |  |
| 60-69 | 235700 | 386(0.16) | 7694(3.26) | 2.87(2.03-4.05) | <0.0001 | 53777 | 89(0.17) | 1715(3.19) | - |  |
| >=70 | 191525 | 664(0.35) | 23159(12.09) | 4.86(3.43-6.87) | <0.0001 | 27684 | 95(0.34) | 2922(10.55) | - |  |
| Other antidiabetic drug |  |  |  |  |  |  |  |  |  |  |
| Insulin | 172662 | 781(0.45) | 25037(14.5) | 3.56(3.15-4.01) | <0.0001 | 57593 | 179(0.31) | 4869(8.45) | 3.41(2.65-4.40) | <0.0001 |
| Metformin | 572714 | 937(0.16) | 21695(3.79) | 0.75(0.67-0.85) | <0.0001 | 182856 | 240(0.13) | 4820(2.64) | 0.89(0.65-1.20) | 0.4345 |
| SU | 198212 | 441(0.22) | 10760(5.43) | 1.09(0.96-1.23) | 0.1859 | 73327 | 131(0.18) | 2590(3.53) | 1.25(0.97-1.62) | 0.0912 |
| DPP-4 inhibitors | 170748 | 417(0.24) | 13830(8.1) | 0.86(0.76-0.98) | 0.0193 | 58139 | 111(0.19) | 2933(5.04) | 0.94(0.73-1.21) | 0.6346 |
| Comorbidity |  |  |  |  |  |  |  |  |  |  |
| Hyperlipidemia | 430723 | 543(0.13) | 10651(2.47) | 0.77(0.69-0.86) | <0.0001 | 138262 | 147(0.11) | 2562(1.85) | - |  |
| HTN | 442260 | 848(0.19) | 25122(5.68) | 0.91(0.82-1.02) | 0.1027 | 122274 | 174(0.14) | 4450(3.64) | - |  |
| CVD | 69945 | 226(0.32) | 9437(13.49) | 1.04(0.88-1.22) | 0.6618 | 14816 | 39(0.26) | 1265(8.54) | - |  |
| COPD | 29454 | 178(0.6) | 5591(18.98) | 1.52(1.27-1.81) | <0.0001 | 5582 | 29(0.52) | 717(12.84) | - |  |
| Asthma | 23629 | 79(0.33) | 1861(7.88) | 1.33(1.05-1.69) | 0.0193 | 5870 | 14(0.24) | 302(5.14) | - |  |
| CKD | 49488 | 196(0.4) | 7383(14.92) | 1.24(1.04-1.48) | 0.0161 | 12146 | 36(0.3) | 1024(8.43) | - |  |
| CCI group |  |  |  |  |  |  |  |  |  |  |
| 0 | 559794 | 797(0.14) | 13751(2.46) | Ref. |  | 169140 | 197(0.12) | 3081(1.82) | Ref. |  |
| 1-2 | 198990 | 449(0.23) | 13674(6.87) | 1.02(0.89-1.16) | 0.7968 | 49680 | 82(0.17) | 2237(4.5) | 0.82(0.62-1.09) | 0.1650 |
| ≧3 | 47594 | 190(0.4) | 10064(21.15) | 1.04(0.86-1.25) | 0.6864 | 9648 | 28(0.29) | 1510(15.65) | 0.89(0.58-1.37) | 0.5927 |
| DCSI group |  |  |  |  |  |  |  |  |  |  |
| 0 | 508438 | 726(0.14) | 14256(2.8) | Ref. |  | 129373 | 121(0.09) | 2192(1.69) | - |  |
| 1 | 147050 | 256(0.17) | 5948(4.04) | 0.93(0.80-1.08) | 0.3316 | 75517 | 130(0.17) | 2780(3.68) | - |  |
| ≧2 | 150890 | 454(0.3) | 17285(11.46) | 0.96(0.82-1.11) | 0.5672 | 23578 | 56(0.24) | 1856(7.87) | - |  |

Data are numbers (percentage) or hazard ratio (95% confidence interval).

Abbreviation: TB, tuberculosis; DM, diabetes mellitus; HR, hazard ratio; AHR, adjusted hazard ratio; CI, confidence interval; SGLT2i, sodium-glucose cotransporter 2 inhibitors; SU, sulfonylureas; DPP-4, dipeptidyl peptidase-4; HTN, hypertension; CVD, cardiovascular disease; COPD, chronic obstructive pulmonary disease; CKD, chronic kidney disease; CCI; Charlson Comorbidity Index; DCSI, Diabetes Complications Severity Index;

**Table S3.** Sensitivity analysis excluding patients receiving other antidiabetic medications during the follow up periods in the SGLT2i group

|  | AHR (95% CI) | p-value |
| --- | --- | --- |
| SGLT2i | 0.37(0.21-0.65) | 0.0006 |
| Non-SGLT2i | Ref. |  |
| CCI group |  |  |
| 0 | Ref. |  |
| 1-2 | 1.02(0.76-1.37) | 0.8920 |
| ≧3 | 1.50(0.98-2.28) | 0.0607 |
| Other antidiabetic drug |  |  |
| Insulin | 3.23(2.45-4.28) | <0.0001 |
| Metformin | 0.79(0.59-1.07) | 0.1263 |
| SU | 1.26(0.95-1.68) | 0.1156 |
| DPP-4 | 0.92(0.39-1.24) | 0.5882 |

Data are numbers (percentage) or hazard ratio (95% confidence interval).

Abbreviation: AHR, adjusted hazard ratio; CI, confidence interval; SGLT2i, sodium-glucose cotransporter 2 inhibitors; CCI; Charlson Comorbidity Index; SU, sulfonylureas; DPP-4, dipeptidyl peptidase-4;

**Table S4.** Sensitivity analysis using SGLT2i initiation date as the index date

|  | Total | TB | Death | AHR (95% CI) | p-value |
| --- | --- | --- | --- | --- | --- |
| SGLT2i initiation date as the index date |  |  |  |  |  |
| SGLT2i | 76159 | 75(0.10) | 1786(2.35) | 0.75(0.57-0.99) | 0.0481 |
| Non-SGLT2i | 150446 | 157(0.10) | 3409(2.27) | Ref. |  |
| Early initiation* |  |  |  |  |  |
| SGLT2i | 40420 | 44(0.11) | 1053(2.61) | 0.60(0.42-0.86) | 0.0050 |
| Non-SGLT2i | 80840 | 128(0.16) | 2784(3.44) | Ref. |  |

Data are numbers (percentage) or hazard ratio (95% confidence interval).

Abbreviation: TB, tuberculosis; AHR, adjusted hazard ratio; CI, confidence interval; SGLT2i, sodium-glucose cotransporter 2 inhibitors;

*Early initiation was defined as patients who initiated SGLT2i use within the first year after DM diagnosis.
